# Randomized phase II trial of FOLFIRI-panitumumab compared with FOLFIRI alone in patients with *RAS* wild-type circulating tumor DNA metastatic colorectal cancer beyond progression to first-line FOLFOX-panitumumab: the BEYOND study (GEMCAD 17-01)

**DOI:** 10.1007/s12094-022-02868-x

**Published:** 2022-06-27

**Authors:** Jorge Aparicio, Anna C. Virgili Manrique, Jaume Capdevila, Félix Muñoz Boza, Patricia Galván, Paula Richart, Helena Oliveres, David Páez, Jorge Hernando, Sara Serrano, Ruth Vera, Xavier Hernandez-Yagüe, Rafael Álvarez Gallego, M. Carmen Riesco-Martinez, Xavier García de Albeniz, Joan Maurel

**Affiliations:** 1 Medical Oncology Department, Hospital Universitari i Politècnic La Fe, Avda. Abril Martorell 106, 46026 Valencia, Spain; 2 Medical Oncology Department, Hospital de la Santa Creu i Sant Pau, Barcelona, Spain; 3 Medical Oncology Department, Vall d’Hebron University Hospital, Vall Hebron Institute of Oncology (VHIO), Barcelona, Spain; 4 IOB-Teknon, Barcelona, Spain; 5 Medical Oncology Department, Hospital Universitari Sant Joan de Reus, Reus, Spain; 6 Translational Genomics and Targeted Therapies in Solid Tumors, Medical Oncology Department, Hospital Clínic de Barcelona, C/ Villaroel, 170, 08036 Barcelona, Spain; 7 Medical Oncology Department, Complejo Hospitalario de Navarra, Pamplona, Spain; 8 Medical Oncology Department, ICO, Girona, Girona, Spain; 9 Medical Oncology Department, Hospital Madrid Norte San Chinarro-Centro Integral Oncologico Clara Campal, Madrid, Spain; 10 Medical Oncology Department, Hospital Universitario, 12 Octubre, Madrid, Spain; 11 RTI Health Solutions, Barcelona, Spain

**Keywords:** Colorectal cancer, Metastatic disease, Second line therapy, Panitumumab, Liquid biopsy

## Abstract

**Purpose:**

Panitumumab plus FOLFOX (P-FOLFOX) is standard first-line treatment for *RAS* wild-type (WT) metastatic colorectal cancer. The value of panitumumab rechallenge is currently unknown. We assessed addition of panitumumab to FOLFIRI (P-FOLFIRI) beyond progression to P-FOLFOX in patients with no *RAS* mutations in liquid biopsy (LB).

**Methods:**

In this randomized phase II trial, patients were assigned (3:2 ratio) to second-line P-FOLFIRI (arm A) or FOLFIRI alone (arm B). LB for circulating tumor DNA analysis was collected at study entry and at disease progression. Primary endpoint was 6-month progression-free survival. Two-stage Simon design required 85 patients to be included (EudraCT 2017-004519-38).

**Results:**

Between February 2019 and November 2020, 49 patients were screened (16 RAS mutations in LB detected) and 31 included (18 assigned to arm A and 13 to arm B). The study was prematurely closed due to inadequate recruitment. Serious adverse events were more frequent in arm A (44% vs. 23%). Overall response rate was 33% (arm A) vs. 7.7% (arm B). Six-month progression-free survival rate was 66.7% (arm A) and 38.5% (arm B). Median progression-free survival was 11.0 months (arm A) and 4.0 months (arm B) (hazard ratio, 0.58). At disease progression, *RAS* or *BRAF* mutations in LB were found in 4/11 patients (36%) in arm A and 2/10 (20%) in arm B.

**Conclusions:**

The BEYOND study suggests a meaningful benefit of P-FOLFIRI beyond progression to P-FOLFOX in metastatic colorectal cancer patients with WT *RAS* status selected by LB. This strategy deserves further investigation.

## Introduction

Colorectal cancer (CRC) represents 12.7% of all new tumors in the European Union (EU) population [1]. Up to 50% of patients will develop metastases during their evolution. For those with unresectable metastatic disease, treatment is mainly palliative and median survival time is about 30 months. So far, only a few therapeutic agents have shown efficacy in metastatic disease: fluoropyrimidines, oxaliplatin, irinotecan, anti-epidermal growth factor receptor (anti-EGFR) drugs as cetuximab and panitumumab, and anti-angiogenic drugs. For fit patients, they are frequently used upfront in combination, i.e., a chemotherapy regimen plus a targeted agent (according to their molecular profile) [2–4]. After progression, second-line regimens include chemotherapy crossover with or without targeted agents. However, treatment options in this setting have limited benefit, and thus, further clinical investigation is warranted.

Activating mutations in *KRAS* and *NRAS* are predictive biomarkers of resistance to anti-EGFR monoclonal antibodies in metastatic CRC. In addition, mutations occurring in *BRAF*, *PIK3CA,* and *PTEN* genes have been associated with resistance to these drugs. Panitumumab is labeled in EU in combination with FOLFOX (fluorouracil, folinic acid, and oxaliplatin) in first line (P-FOLFOX) [5] and with FOLFIRI (fluorouracil, folinic acid, and irinotecan) in second line (P-FOLFIRI) [6] for *RAS* wild-type (WT) patients. The clinical value of anti-EGFR rechallenge strategy (i.e., re-treat with the anti-EGFR in patients previously treated with anti-EGFR and with progressive disease to a second line) has not been definitively demonstrated in randomized clinical trials, although some evidence suggests that it could have potential value [7–10]. In a somewhat different approach, Ciardello et al. tested for the first time cetuximab continuation plus chemotherapy crossover beyond first progression and showed its potential therapeutic efficacy in molecularly selected patients (*KRAS*, *NRAS*, *BRAF*, and *PIK3CA* WT status, assessed in baseline tumor biopsy by next-generation sequencing) [7].

Plasma circulating tumor DNA (ctDNA) determination of *RAS* and *BRAF* has been shown to correlate well with tissue determination in multiple studies with different techniques [11–14]. Therefore, continuous monitoring of *RAS* status is useful during the course of the disease, because it avoids the inconveniences of repeating tumor tissue samples biopsies and is more representative of the current mutational status of the disease. Prospective and retrospective cohort studies suggest that ctDNA *RAS* mutations detected by liquid biopsy (LB) can identify metastatic colorectal cancer patients with intrinsic or acquired anti-EGFR resistance [15–17]. Recently, Cremolini et al. suggested in a retrospective analysis of a prospective phase II clinical trial [18] that a rechallenge strategy with cetuximab benefits specially patients who maintain *RAS* and *BRAF* WT status in LB.

We aimed to explore the clinical activity of maintaining panitumumab in combination with FOLFIRI beyond progression to first-line P-FOLFOX in patients with no *RAS* mutations detected before second-line treatment by LB technology.

## Methods

### Study design and patients

BEYOND trial (GEMCAD 17-01) is an academic, open-label, randomized phase II trial performed at 15 Spanish hospitals. This trial is registered with EudraCT, no.: 2017-004519-3 8. We enrolled patients aged 18 years or older with histologically confirmed adenocarcinoma of colon or rectum, measurable metastatic disease not amenable to surgical resection, confirmed disease progression to first-line treatment according to RECIST criteria (version 1.1), ECOG performance status of 0–2, and adequate bone marrow, liver and renal function. Patients should have been previously treated in first line with P-FOLFOX, should have achieved complete response (CR), partial response (PR), or stable disease (SD), and have ctDNA WT *RAS* status determined before randomization. The main exclusion criteria were relevant cardiovascular disease, central nervous system metastases, unresolved toxicities of previous systemic treatment, acute or subacute intestinal occlusion, active inflammatory bowel disease, and major surgery or radiotherapy within 28 days prior to inclusion in the study. The trial was approved by the Ethics Committees at each participating institution and was carried out in accordance with the Declaration of Helsinki. All patients provided written informed consent.

### Randomization and treatment

LB for ctDNA analysis was collected at study entry and at disease progression with Idyilla technology. After confirmed progression to first- line P-FOLFOX and having verified the absence of mutations in ctDNA, patients were centrally randomized in a 3:2 ratio to P-FOLFIRI (arm A) or FOLFIRI alone (arm B). Random assignment was stratified by primary tumor sidedness (left vs. right) and, after an amendment dated January 20, 2020, by time since last panitumumab administration (≤ 3 months vs. > 3 months).

On day 1 of each 14-day period, patients in the FOLFIRI group received an infusion of 200 mg l-folinic acid over 1.5–2 h(s), 180 mg per square meter of irinotecan over 1.5 h, 400 mg per square meter bolus of 5-fluorouracil (5-FU) and then 46-h continuous infusion (CI) of 2400 mg per square meter of 5-fluorouracil; patients in the P-FOLFIRI group received an infusion of panitumumab 6 mg per kilogram over 60 min before FOLFIRI. Those patients ≥ 70 years old could start FOLFIRI with a 20% dose reduction. Treatment was continued until disease progression, unacceptable toxic effects, or withdrawal of consent occurred.

Computed tomography imaging was performed at baseline and every 8 weeks until disease progression. Tumor response was evaluated according to Response Evaluation Criteria in Solid Tumors (RECIST, version 1.1). Adverse events [assessed according to the National Cancer Institute-Common Toxicity Criteria for adverse events (AEs), version 4.03] were recorded continuously.

### Statistical analysis

The primary endpoint was 6-month progression-free survival (PFS), defined as the proportion of subjects still alive and progression free at 6 months, and analyzed by intention to treat. The aim of the control arm was to test the validity of the assumption of null effect (6-month PFS of 30%). Sample size was determined through a Simon's two-stage model assuming a null effect corresponding to a 6-month PFS of 30% and a treatment effect corresponding to a 6-month PFS of 50%, with an alpha error of 0.05, and a beta error of 0.2. The sample needed for this study was 46 evaluable subjects in arm A. Considering 10% of possible losses, the final patient number projected to be included was 51 in arm A and 34 in arm B. Under this design, an interim analysis was planned 6 months after the inclusion of the first 15 subjects in arm A and, if the number of patients without progression at 6 months in arm A was less than or equal to 5, the trial would be stopped prematurely because of futility. Analysis of PFS at 6 months was based on the Kaplan–Meier estimator. The survival function as well as the median [95% confidence interval -95% CI-] time to event were estimated by means of the Kaplan–Meier method. Group comparisons were done using the (stratified) log-rank test and the (stratified) hazard ratios (95% CI) were estimated with the Cox model. Secondary analyses were overall response rate (ORR), overall survival (OS), safety and tolerability, as well as biomarker analysis by tissue and LB.

## Results

### Patients

**Fig. 1:**
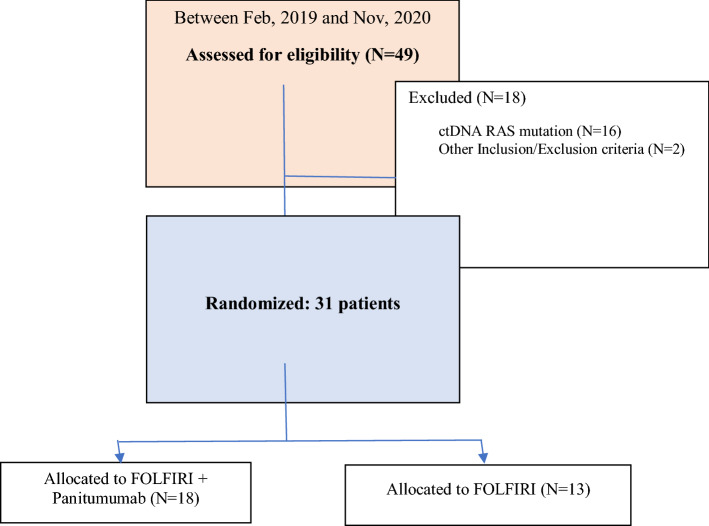
Study flowchart

From February 2019 through November 2020, 49 patients were screened for *RAS/BRAF/PI3K* mutations in ctDNA prior to enrollment. Eighteen patients were excluded by not meeting inclusion/exclusion criteria: sixteen patients because of RAS mutations (32.6%), one patient because not having achieved at least stable disease to first-line P-FOLFOX and one patient due to CNS metastasis at screening. Of 31 eligible patients, 18 were randomized to arm A and 13 to arm B (Fig. 1). Interim analysis was conducted as planned, showing 6 patients without progression 6 months after the inclusion of 15 patients in arm A; therefore, the study continued. However, it was prematurely closed due to low recruitment.

Baseline characteristics were well balanced across study arms (Table 1). There were no major differences in time from the last dose of first-line chemotherapy, time from first-line panitumumab to randomization or in the duration of panitumumab in previous line. Median relative dose intensity for irinotecan, bolus 5-FU, and CI 5-FU was similar in both arms. Median treatment duration and median number of subjects with dose reductions and dose interruptions were higher in arm A. Median number of cycles administered was 12 (Interquartile Range, IQR 7–17) for arm A and 6 (IQR 5–9) for arm B. The only imbalances corresponded to performance status (ECOG PS), with more patients in arm B having ECOG PS 1 (76.9% vs 44.4% in Arm A) and more than one organ affected (100% vs. 55.6%). Altogether, most patients were male, 42% had an ECOG PS of 0, left colon was the primary tumor site in 77.4%, and metastases were located mainly in the liver and lungs. The median duration of follow-up was 9.5 months (IQR 6.0–13.0) with P-FOLFIRI and 7 months (IQR, 5.0–9.0) with FOLFIRI.

**Table 1:** Baseline patients and disease characteristics

		Group A (*N* = 18)	Group B (*N* = 13)	Total (*N* = 31)
Age (years)				
	Median [Q1–Q3]	59 [51–66]	67 [62–74]	62 [55–73]
	Min–Max	31–78	55–84	31–84
Gender				
Female	*n* (%)	8 (44.4)	3 (23.1)	11 (35.5)
Male	*n* (%)	10 (55.6)	10 (76.9)	20 (64.5)
Primary tumor location				
Left side	*n* (%)	13 (72.2)	11 (84.6)	24 (77.4)
Right side	*n* (%)	5 (27.8)	2 (15.4)	7 (22.6)
ECOG				
ECOG 0	*n* (%)	10 (55.6)	3 (23.1)	13 (41.9)
ECOG 1	*n* (%)	8 (44.4)	10 (76.9)	18 (58.1)
CEA (ng/ml)				
	Median	11.0 [1.5–881.0]	20.6 [2.9–171.0]	16.6 [1.5–881.0]
LDH (uKat/L)				
	Median	5.55 [3.7, 8.5]	5.99 [2.9, 7.6]	5.61[3.5, 8.2]
Metastases				
Liver metastases	*n* (%)	14 (77.8)	10 (76.9)	24 (77.4)
Lymph-node metastases	*n* (%)	5 (27.8)	9 (69.2)	14 (45.2)
Lung metastases	*n* (%)	9 (50.0)	7 (53.8)	16 (51.6)
Peritoneum metastases	*n* (%)	4 (22.2)	4 (30.8)	8 (25.8)
Other metastases	*n* (%)	2 (11.1)	1 (7.7)	3 (9.7)
Number of organs affected				
1	*n* (%)	8 (44.4)	0 (0.0)	8 (25.8)
2	*n* (%)	5 (27.8)	8 (61.5)	13 (41.9)
3 or more	*n* (%)	5 (27.8)	5 (38.5)	10 (32.3)
Time from last dose of panitumumab to randomization (months)				
	Mean (range)	2.67 (1.0–7.0)	2.15 (1.0–6.0)	2.45 (1.0–7.0)
Time from last dose of first-line chemotherapy to randomization (months)				
	Mean (range)	4.61 (1.0–18.0)	2.38 (1.0–6.0)	3.68 (1.0–18.0)
Duration of panitumumab in first line				
Less than 3 months	*n* (%)	0 (0.0)	2 (15.4)	2 (6.5)
3 months or more	*n* (%)	18 (100)	11 (84.6)	29 (93.5)

### Efficacy

**Fig. 2:**
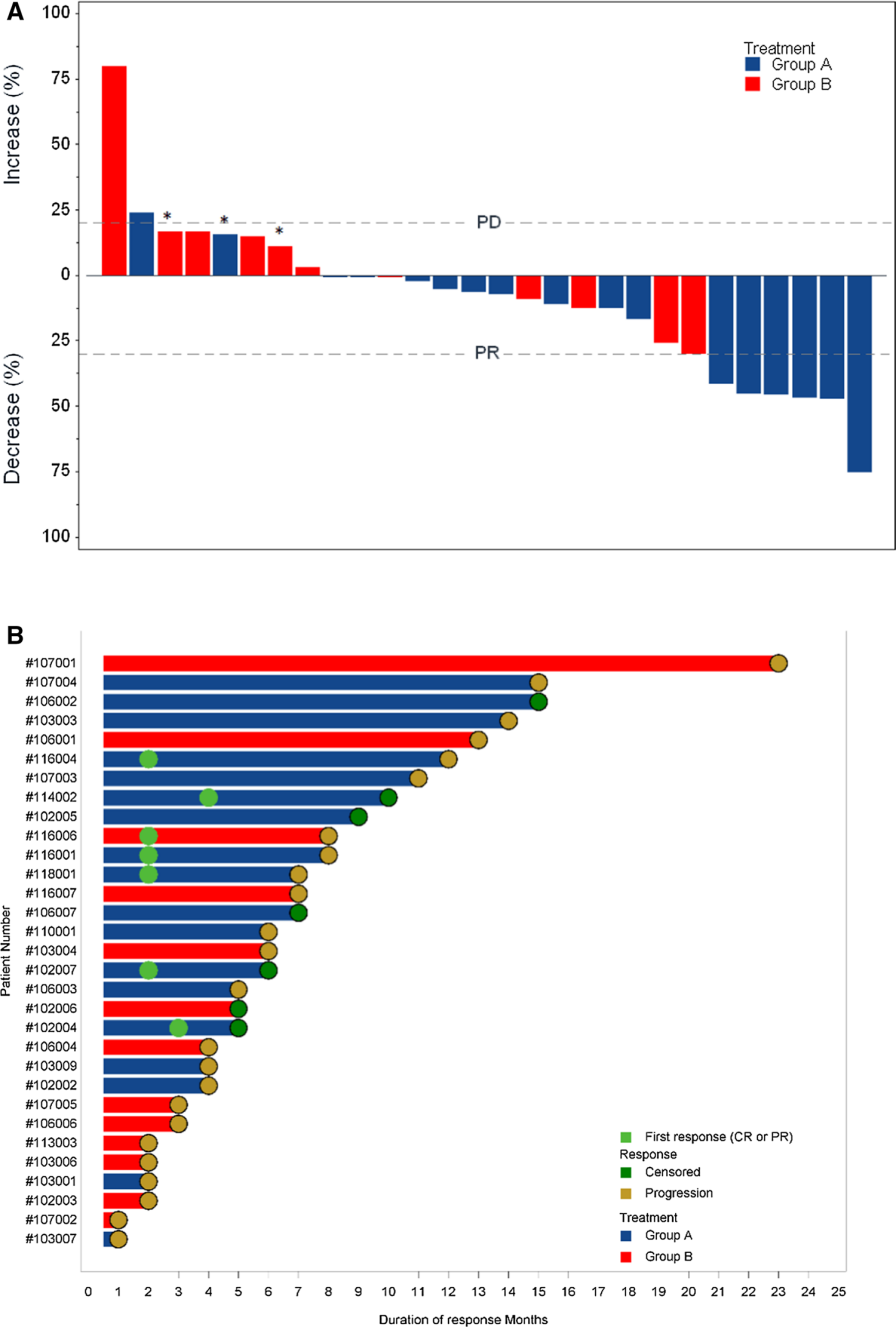
Radiologic response. **A** Waterfall plot of tumor response in evaluable patients, by treatment group. The bars show the best percentage change in the target lesions from baseline. The dashed horizontal lines at 20% and − 30% represent the progressive disease and partial response, respectively. *Patients with progression disease (PD) as best overall response due to new target lesions. **B** Swimmer plot showing time of objective response (PR or CR) and treatment duration

**Fig. 3:**
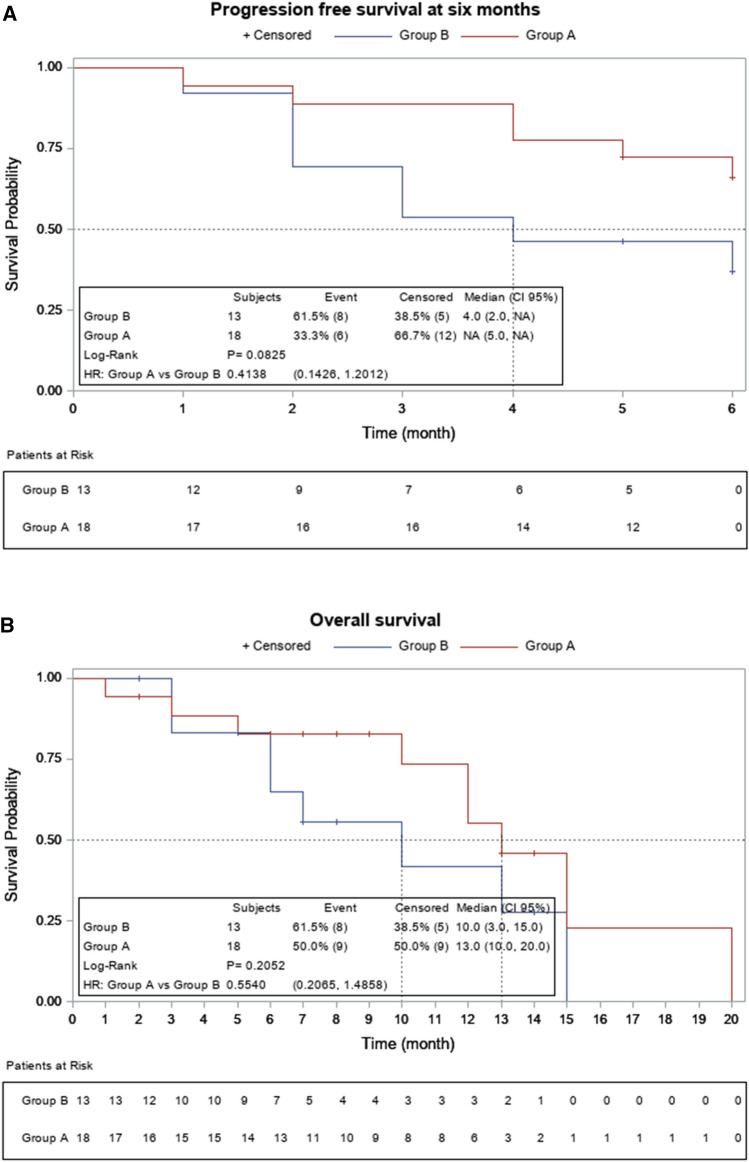
Survival curves. Kaplan–Meier curve for progression-free survival (**A**). Kaplan–Meier curve for overall survival (**B**)

One patient in each arm was not evaluable for response. Confirmed partial tumor responses occurred in 6 patients (33%; 95% confidence interval CI 13–59) in arm A and in 1 patient (7.7%; 95% CI 0.2–36) in arm B. Disease stabilizations were seen in 9 (50%) and 7 (54%) patients, respectively, for a disease control rate of 83% in arm A and 62% in arm B. No complete responses were observed. Best overall response and time of objective response are depicted in Fig. 2. Six-month PFS was 66.7% (95% CI 40.9–86.6) in arm A and 38.5% (95% CI 13.8–68.4) in arm B, corresponding to a hazard ratio (HR) of 0.42, (95% CI 0.14–1.23) (Fig. 3A). The median PFS was 11 months (95% CI 4–14) for arm A and 4 months (95% CI 2–8) for arm B (HR 0.58, 95% CI 0.25–1.3). Median overall survival was 13 months (95% CI 10–20) and 10 months (CI 95% 3–15) in arms A and B, respectively, corresponding to a HR of 0.55 (95% CI 0.2–1.48) (Fig. 3B). At disease progression, mutational status by LB was determined in 21 out of 24 patients, and emergent mutations in *RAS* were found in 4 out 11 patients (36%) in arm A and in 2 out of 10 patients (20%) in arm B (Table 2). There was a BRAF mutational status change from mutated to wild type in arm B.

**Table 2:** Type of ctRAS mutation at progression

Patient	ctKRAS results	Type of mutation	ctNRAS results	Type of mutation
#P1	Mutation detected in KRAS CODON 61	Q61H	No mutation	
#P2	Mutation detected in KRAS CODON 61	Q61H	No mutation	
#P3	Mutation detected in KRAS CODON 12	G12D	No mutation	
#P4	Mutation detected in KRAS CODON 12	G12C	No mutation	
#P5	Mutation detected in KRAS CODON 61	Q61H	Mutation detected in NRAS CODON 61	Q61R/K
#P6	Mutation detected in KRAS CODON 61	Q61H	No mutation	

### Adverse events

All patients in the safety population presented at least one adverse event. Overall incidence of grade 3 or 4 adverse events was 66.7% in the P-FOLFIRI group and 53.9% in the FOLFIRI group. The administration of P-FOLFIRI, as compared with FOLFIRI alone, was associated with more asthenia (50% vs. 38.5%), hypomagnesemia (38.9% vs. 7.7%), and acneiform rash (38.9% vs. 0.0%), all of them mainly grade 1–2. With P-FOLFIRI, there was a higher incidence of grade 3 diarrhea (16.7% vs. 7.7%), and in the FOLFIRI arm, there was more frequency of grade 3–4 anemia (15.4% vs 5.6%) (Table 3). Serious AEs were experienced by 44.4% of patients who received P-FOLFIRI compared with 23.1% of patients treated with FOLFIRI. There were two AEs leading to death in both treatment arms (one case in each arm); however, they were not judged as treatment-related by the study investigators.

**Table 3:** Most common adverse events in the safety population (> 10% incidence in either treatment arm)

SOC/PT	Group A				Group B			
	Grade 1 and 2	Grade 3 and 4	Grade 5	Total	Grade 1 and 2	Grade 3 and 4	Grade 5	Total
	Subjects *n* (%)	Subjects *n* (%)	Subjects *n* (%)	Subjects *n* (%)	Subjects *n* (%)	Subjects *n* (%)	Subjects *n* (%)	Subjects *n* (%)
Overall	18 (100.0)	12 (66.7)	1 (5.6)	18 (100.0)	11 (84.6)	7 (53.9)	1 (7.7)	12 (92.3)
Anemia	6 (33.3)	1 (5.6)	0 (0.0)	6 (33.3)	4 (30.8)	2 (15.4)	0 (0.0)	6 (46.2)
Neutropenia	6 (33.3)	4 (22.2)	0 (0.0)	8 (44.4)	5 (38.5)	3 (23.1)	0 (0.0)	6 (46.2)
Constipation	3 (16.7)	0 (0.0)	0 (0.0)	3 (16.7)	4 (30.8)	1 (7.7)	0 (0.0)	4 (30.8)
Diarrhea	11 (61.1)	3 (16.7)	0 (0.0)	11 (61.1)	9 (69.2)	1 (7.7)	0 (0.0)	9 (69.2)
Intestinal perforation	0 (0.0)	0 (0.0)	1 (5.6)	1 (5.6)	0 (0.0)	0 (0.0)	0 (0.0)	0 (0.0)
Asthenia	9 (50.0)	2 (11.1)	0 (0.0)	9 (50.0)	5 (38.5)	1 (7.7)	0 (0.0)	5 (38.5)
Death	0 (0.0)	0 (0.0)	0 (0.0)	0 (0.0)	0 (0.0)	0 (0.0)	1 (7.7)	1 (7.7)
Fatigue	3 (16.7)	0 (0.0)	0 (0.0)	3 (16.7)	1 (7.7)	0 (0.0)	0 (0.0)	1 (7.7)
Mucosal inflammation	5 (27.8)	0 (0.0)	0 (0.0)	5 (27.8)	1 (7.7)	1 (7.7)	0 (0.0)	2 (15.4)
Pyrexia	0 (0.0)	0 (0.0)	0 (0.0)	0 (0.0)	1 (7.7)	1 (7.7)	0 (0.0)	2 (15.4)
Folliculitis	2 (11.1)	0 (0.0)	0 (0.0)	2 (11.1)	0 (0.0)	0 (0.0)	0 (0.0)	0 (0.0)
Urinary tract infection	2 (11.1)	0 (0.0)	0 (0.0)	2 (11.1)	0 (0.0)	0 (0.0)	0 (0.0)	0 (0.0)
Alanine aminotransferase increased	2 (11.1)	0 (0.0)	0 (0.0)	2 (11.1)	0 (0.0)	0 (0.0)	0 (0.0)	0 (0.0)
Blood bilirubin increased	1 (5.6)	1 (5.6)	0 (0.0)	2 (11.1)	0 (0.0)	0 (0.0)	0 (0.0)	0 (0.0)
Platelet count decreased	1 (5.6)	1 (5.6)	0 (0.0)	2 (11.1)	0 (0.0)	0 (0.0)	0 (0.0)	0 (0.0)
Decreased appetite	4 (22.2)	0 (0.0)	0 (0.0)	4 (22.2)	5 (38.5)	0 (0.0)	0 (0.0)	5 (38.5)
Hypokalaemia	0 (0.0)	2 (11.1)	0 (0.0)	2 (11.1)	0 (0.0)	0 (0.0)	0 (0.0)	0 (0.0)
Hypomagnesaemia	6 (33.3)	1 (5.6)	0 (0.0)	7 (38.9)	1 (7.7)	0 (0.0)	0 (0.0)	1 (7.7)
Back pain	2 (11.1)	0 (0.0)	0 (0.0)	2 (11.1)	1 (7.7)	0 (0.0)	0 (0.0)	1 (7.7)
Dysphonia	2 (11.1)	0 (0.0)	0 (0.0)	2 (11.1)	0 (0.0)	0 (0.0)	0 (0.0)	0 (0.0)
Dyspnea	3 (16.7)	0 (0.0)	0 (0.0)	3 (16.7)	0 (0.0)	0 (0.0)	0 (0.0)	0 (0.0)
Alopecia	3 (16.7)	0 (0.0)	0 (0.0)	3 (16.7)	1 (7.7)	0 (0.0)	0 (0.0)	1 (7.7)
Erythema	2 (11.1)	1 (5.6)	0 (0.0)	3 (16.7)	0 (0.0)	0 (0.0)	0 (0.0)	0 (0.0)
Onycholysis	2 (11.1)	0 (0.0)	0 (0.0)	2 (11.1)	0 (0.0)	0 (0.0)	0 (0.0)	0 (0.0)
Pruritus	2 (11.1)	0 (0.0)	0 (0.0)	2 (11.1)	0 (0.0)	0 (0.0)	0 (0.0)	0 (0.0)
Rash	7 (38.9)	2 (11.1)	0 (0.0)	7 (38.9)	0 (0.0)	0 (0.0)	0 (0.0)	0 (0.0)
Skin toxicity	4 (22.2)	1 (5.6)	0 (0.0)	4 (22.2)	0 (0.0)	0 (0.0)	0 (0.0)	0 (0.0)
Vascular disorders	2 (11.1)	0 (0.0)	0 (0.0)	2 (11.1)	0 (0.0)	0 (0.0)	0 (0.0)	0 (0.0)

Percentages are based on the number of subjects (*N*) in a given study group or overall as the denominator

For each row category, a subject with two or more adverse events in that category is counted only once for the patients column

System Organ Class and Preferred Term are based on the Version 18.0 of the MedDRA dictionary

*Events* number of events, *Subjects* number of subjects

*Group A* FOLFIRI + panitumumab Therapy, *Group B* FOLFIRI alone Therapy

## Discussion

In the continuum of care of metastatic CRC patients treated in first line with chemotherapy plus the anti-vascular endothelial growth factor bevacizumab, it has been shown that a therapeutic option is second-line chemotherapy in combination with anti-angiogenic drugs (bevacizumab, aflibercept, or ramucirumab). It suggests that anti-angiogenic therapy beyond first progression can be effective [21–24]. For *RAS* WT patients, anti-EGFR therapy (either alone or in combination with chemotherapy) is considered standard in only a single line of therapy (i.e., first, second, or third) for non-pre-treated patients. More recently, in the setting of anti-EGFR therapy after a first-line anti-EGFR-containing regimen, two different experimental approaches have been explored: (1) the rechallenge, i.e., reintroduction of anti-EGFR therapy after a second-line, anti-EGFR-free schedule; and (2) the continuation of anti-EGFR therapy (plus chemotherapy crossover) beyond progression to an upfront regimen. Both approaches have been assessed with promising results in phase II trials [7, 18, 26]. The underlying hypothesis is that a sustained inhibition of EGFR signaling would continuously eliminate sensitive clones of RAS WT tumor. Pending phase III confirmation, all these studies suggested that the evaluation of RAS mutational status on ctDNA might be helpful in selecting candidate patients. Only patients with *RAS* and *BRAF* WT status at relapse determined by LB seem to benefit from these second- or third-line therapies [15].

The BEYOND trial shows a potential benefit of adding panitumumab to FOLFIRI, in comparison with FOLFIRI alone, as second-line treatment for patients with ctDNA WT *RAS* status. Unfortunately, the estimates are imprecise because of the low number of patients recruited. This is a novel approach evaluating the effects of continuing EGFR inhibition for metastatic CRC beyond tumor progression to a first-line panitumumab-containing treatment. Although the calculated sample size of the study was not met, our results do not rule out a beneficial effect of this re-treatment strategy. Our final figures suggest that we would need 140 patients to be included in a phase III clinical trial to perform a positive study.

The ctDNA study included in our trial confirms data from previous studies [19, 20], indicating that roughly two-thirds of patients receiving anti-EGFR schedules retain their *RAS* WT status at disease progression. These are accurately the patients that most likely benefit from anti-EGFR re-treatment. ORR (between 6 and 10%) and PFS (between 4 and 5 months) in our control arm are within the range of those reported in the literature with second-line FOLFIRI [21–23] or FOLFIRI plus bevacizumab [24] and for the *RAS* naïve enriched population treated with FOLFIRI [6]. Therefore, the observed differences are unlikely to arise from a worse outcome in the control arm. We decided to use FOLFIRI alone, instead of FOLFIRI plus bevacizumab in the control arm, because there is no clinical evidence that bevacizumab provides benefit compared to FOLFIRI based on phase III clinical trials after first-line P-FOLFOX. Recently, a prospective clinical trial comparing second-line FOLFIRI plus cetuximab vs FOLFIRI plus bevacizumab in *RAS* WT (based on baseline tumor tissue determination) metastatic CRC patients previously treated with FOLFOX plus cetuximab did not demonstrate cetuximab benefit in response rate or PFS [25], but suggested that only patients that retain *RAS* WT status before second-line therapy would benefit from rechallenge strategy [18]. Our ORR (33%; 95% CI 13–59%) and PFS (11 months; 95% CI 4–14 months) in the experimental arm are in line with that obtained with second-line P-FOLFIRI in FOLFOX only pre-treated patients [6]. Recently, the CHRONOS study [26] with a rechallenge strategy showed similar ORR (30%, 95% CI 12–47%) than that achieved with panitumumab monotherapy in previously untreated patients [27].

Despite initial benefit in the P-FOLFIRI arm, most patients included in the study progressed. Interestingly, at the time of disease progression, only 36% of patients in the P-FOLFIRI group showed emergent *RAS* mutations in LB, which is in the range of other studies [28]. We have observed *RAS* emergent mutations in 20% of patients of the FOLFIRI control arm, suggesting that acquisition of *RAS* mutations could appear also on holding anti-EGFR periods. All these data confirm that alternative driven mutations in *EGFR*, *PIK3CA*, *AKT*, or ERBB2, MET amplification [29–34], or transcriptomic mechanisms of resistance [35] play a critical role in intrinsic and acquired anti-EGFR resistance.

The safety profile of P-FOLFIRI treatment was in line with that expected. The incidence rate of grade 3 or diarrhea and acne-like rash reactions were higher with P-FOLFIRI than with FOLFIRI alone, as well as the overall grade 3 or 4 adverse events. However, these adverse events were generally manageable. Regrettably, we did not performed quality of life questionnaires that would have better assessed the clinical impact of this treatment.

In conclusion, the BEYOND study suggests a meaningful clinical benefit of adding panitumumab to FOLFIRI beyond progression to first-line P-FOLFOX in metastatic CRC patients with WT *RAS* status selected by LB at relapse. More globally, it also supports the potential value of these novel approaches evaluating anti-EGFR re-treatment (continuation or rechallenge) in the second- or third-line treatment. Although the study was closed prematurely, this strategy deserves further investigation.
